# Benign ethnic neutropenia in a South African population, and its association with HIV acquisition and adverse event reporting in an HIV vaccine clinical trial

**DOI:** 10.1371/journal.pone.0241708

**Published:** 2021-01-22

**Authors:** Rephaim Mpofu, Kennedy Otwombe, Koleka Mlisana, Maphoshane Nchabeleng, Mary Allen, James Kublin, M. Juliana McElrath, Linda-Gail Bekker, Gavin Churchyard, Glenda Gray, Fatima Laher

**Affiliations:** 1 Faculty of Health Sciences, Perinatal HIV Research Unit, University of Witwatersrand, Johannesburg, South Africa; 2 Division of Clinical Pharmacology, Department of Medicine, University of Cape Town, Cape Town, South Africa; 3 Faculty of Health Sciences, School of Public Health, University of the Witwatersrand, Johannesburg, South Africa; 4 National Health Laboratory Service (NHLS), Cape Town, South Africa; 5 Centre for the AIDS Programme of Research in South Africa (CAPRISA), Johannesburg, South Africa; 6 Mecru Clinical Research Unit, Sefako Makgatho Health Sciences University, Pretoria, South Africa; 7 National Institute of Allergy and Infectious Diseases, National Institutes of Health, Bethesda, Maryland, United States of America; 8 Vaccine and Infectious Diseases Division, Fred Hutchinson Cancer Research Center, Seattle, Washington, United States of America; 9 Desmond Tutu HIV Centre, University of Cape Town, Cape Town, South Africa; 10 Aurum Institute, Johannesburg, South Africa; 11 South African Medical Research Council, Cape Town, South Africa; IAVI, UNITED STATES

## Abstract

Benign ethnic neutropenia (BEN) is defined as a neutrophil count of <1.5×10^9^ cells/L in healthy individuals and is more common in populations of certain ethnicities, e.g. African or Middle Eastern ethnicity. Neutrophil values are commonly included in eligibility criteria for research participation, but little is known about the relationship between BEN, HIV acquisition, and the occurrence of adverse events during clinical trials. We investigated these relationships using data from an HIV vaccine efficacy trial of healthy adults from 5 South African sites. We analysed data from the double-blind, placebo-controlled, randomized trial HVTN 503, and its follow-on study HVTN 503-S to assess the prevalence of BEN, its association with HIV infection, and adverse event reporting. These data were then compared with a time- and age-matched, non-pregnant cohort from the National Health and Nutrition Examination Survey (NHANES) conducted between 2007–2008 in the United States (US). The 739 South African participants had a median age of 22.0 years (interquartile range = 20–26) and 56% (n = 412) were male. Amongst the US cohort of 845 participants, the median age was 26 (IQR: 21–30) and the majority (54%, 457/745) were also male. BEN was present at enrolment in 7.0% (n = 52) of South African participants (6% in the placebo group versus 8% in the vaccine group); 81% (n = 42) of those with BEN were male. Pretoria North had the highest prevalence of BEN (11.6%, 5/43), while Cape Town had the lowest (0.7%, 1/152). Participants with BEN had a lower median neutrophil count (1.3 vs. 3.2x10^9^ cells/L; p<0.001) and BMI (20.8 vs. 22.3 kg/m^2^; p<0.001) when compared to those without BEN. A greater proportion of Black South Africans had neutrophil counts <1.5×10^9^ cells/L compared to US non-Hispanic Whites from the NHANES cohort (7% [52/739] vs. 0.6% [3/540]; p<0.001). BEN did not increase the odds for HIV infection (adjusted odds ratio [aOR]: 1.364, 95% confidence interval [95% CI]: 0.625–2.976; p = 0.4351). However, female gender (aOR: 1.947, 95% CI: 1.265–2.996; p = 0.0025) and cannabis use (aOR: 2.192, 95% CI: 1.126–4.266; p = 0.0209) increased the odds of HIV acquisition. The incidence rates of adverse events were similar between participants in the placebo group with BEN, and those without: 12.1 (95% CI: 7.3–20.1) vs. 16.5 (95% CI: 14.6–18.7; p = 0.06) events per 100 person-years (py) were noted in the infections and infestations system organ class, respectively. The vaccine group had an event incidence rate of 19.7 (95% CI: 13.3–29.2) vs. 14.8 (95% CI: 13.0–16.8; p = 0.07) events per 100py in the group with, and without BEN, respectively. BEN is more prevalent in Black South Africans compared to US Non-Hispanic Whites. Our data do not support excluding populations from HIV vaccine trials because of BEN. BEN was not associated with increased risk for HIV infection or Adverse events on a vaccine trial. Predictors of HIV infection risk were females and cannabis use, underlying the continued importance of prevention programmes in focusing on these populations.

## Introduction

Africa has a disproportionate HIV burden: in 2019, 54% of the 38 million HIV-infected individuals and 43% of the 1.7 million new HIV infections lived in southern and eastern Africa [1]. Multiple socio-behavioural (including sexual behaviour and drug use) and biological factors (including lack of male circumcision and genetics) have already been shown to be associated with increased HIV acquisition risk [2–6].

An additional, possible contributory factor to the increased HIV prevalence in Africa is the reduced neutrophil count observed in populations of African, Jewish and Arab descent, known as benign ethnic neutropenia (BEN) [4,6]. BEN is a term used to describe decreased absolute neutrophil counts (ANC) amongst healthy individuals from certain ethnic populations without known secondary causes, and not associated with an increased risk of localized or systemic infections [7]. While reference ranges vary among laboratories, and by age, an ANC value of 1.5x10^9^ cells/L is widely used as a lower reference value in clinical practice [7]. This value was partly derived from clinical trials that sought to evaluate the myelosuppressive effects of chemotherapy, and participants involved in these trials were mostly of European origin [8]. Reference ranges previously obtained from non-African populations may, therefore, have limited applicability to populations of African ethnicity in clinical practice or research [9,10]. A previous study demonstrated that an additional 16% of potentially eligible, healthy participants were excluded from participation in a rotavirus vaccine clinical trial on the basis of neutropenia because reference ranges inappropriate for the study population were used in their assessment [11]. The use of inappropriate reference ranges in clinical trials to determine participant eligibility may result in the underrepresentation of this population in research. More recently, practices are shifting toward using locally obtained reference ranges [12,13].

The prevalence of BEN is estimated to range from 2.7–50% across various geographical regions [14–21]. A paper in 2010 reported a decreased mean ANC of approximately 1.0x10^9^ cells/L when comparing an American cohort of non-Hispanic Black males and females with non-Hispanic White males and females [22]. Another study performed in an African population also showed lower neutrophil counts when compared to the neutrophil count distribution from a US-based cohort [23]. The prevalence of BEN in healthy cohorts of African-American lineage in developed countries has previously been documented, but little research has been conducted in the South African population to describe its epidemiology [14,19,24]. It is therefore unknown if the prevalence of BEN in this population is similar to patterns described in other populations that have been associated with BEN.

BEN is not associated with an increased risk of infections [7], but little is known about whether BEN may be associated with an increased frequency of adverse event reporting in clinical trials. Adverse event reporting is critical in the pharmaceutical product development process as it characterizes the safety profile of the investigational product [25]. Additionally, the potential impact of BEN on HIV acquisition or immune responses to investigational HIV vaccines has not been fully assessed. It is possible that population factors such as BEN may influence immune responses to vaccines, or adverse event reporting, which can, in turn, influence the described efficacy and safety profile of the investigational product.

For this study, we investigated: 1) the epidemiology of BEN in various geographical regions among South African vaccine clinical trial participants and compared it with a US cohort, 2) the association between BEN and HIV acquisition, and 3) the reported adverse event rates in the presence or absence of BEN.

## Methods

This is an analysis of data collected from the HVTN 503 (Phambili) trial and its follow-on HVTN 503-S sub-study. HVTN 503 was a phase 2b, multicentre, randomized, double-blinded, placebo-controlled trial that was conducted among sexually active, HIV-1 seronegative participants in South Africa. It aimed to assess the safety and efficacy of the MRKAd5 HIV-1 gag/pol/nef vaccine. The trial was conducted in the Gauteng (Soweto and Pretoria North), North West (Klerksdorp-Orkney-Stilfontein-Hartbeesfontein), KwaZulu-Natal (Durban), and Western Cape (Cape Town) provinces of South Africa. Between January and September 2007, 801 healthy, 18-35-year-old, HIV-uninfected participants were enrolled in HVTN 503 [26]. Enrolment and vaccinations in HVTN 503 were stopped early after non-efficacy of the same product was found at an interim analysis in the HVTN 502 (Step) trial. Participants were unblinded, but safety follow-up was continued [26]. HVTN 503-S was a follow-up study conducted from June 2013 to January 2014 that aimed to assess the HIV acquisition risk of participants enrolled in HVTN 503. The follow-up study enrolled 230 vaccine recipients and 235 placebo recipients. HVTN 503-S found a sustained, increased risk of HIV-1 acquisition among vaccinated men [27]. Methods for HVTN 503 and HVTN 503-S have been described elsewhere [26,27].

This research study was approved by the ethical review committees and institutional review boards of the University of the Witwatersrand, University of Cape Town, University of Limpopo (Medunsa Campus) and the University of KwaZulu-Natal. The clinical trials were approved by the relevant regulatory bodies in the US and South Africa and were also registered on Clinicaltrials.gov (NCT00413725) and the South Africa National Health Research Database (DOH-27-0207). Written informed consent was obtained from the participants at the screening visit and was also confirmed at enrolment and subsequent visits.

HVTN 503 inclusion criteria required that eligible participants be of good general health, which was assessed by a clinical investigator at screening and enrolment. Participants enrolled into the study were therefore judged to be of good general health and unlikely to have any medical conditions that may have influenced the participants’ ANC at the time of haematological assessment. For this analysis, all reported medical conditions that were ongoing at enrolment, and concomitant medication used on the day of enrolment, were re-assessed by qualified medical personnel to identify possible secondary causes of neutropenia that were present at the time of assessment. Possible contributory causes were obtained from a review of published literature [14,16,20]. Participants with neutropenia associated with a possible contributory history of medical conditions or medication use on the day of specimen collection were excluded from the analysis. Complete blood count and HIV infection diagnostic algorithm results, demographics, and behavioural risk data collected on the day of enrolment were obtained from the Statistical Center for HIV/AIDS Research and Prevention. Ethnicity was self-determined by participants at their initial screening visit.

At the screening visit, HIV infection testing was performed using rapid testing kits approved by the Food and Drug Association. Post enrolment, HIV testing was performed according to an in-study HIV diagnostic algorithm, which allowed for differentiation of vaccine-induced sero-positivity from true HIV infection. Full blood count samples were collected on the day of enrolment before vaccination and were analysed using a Beckman Coulter LH500 analyser within 48 hours of collection. Adverse event data collected throughout the main trial was coded according to the Medical Dictionary for Regulatory Activities (version 14.1) classification system [28]. Adverse events were graded during the clinical trial according to the Division of AIDS Table for Grading the Severity of Adult and Pediatric Adverse Events, Version 1.0, December, 2004 [29].

### Statistical analysis

Neutropenia was defined as an ANC <1.5x10^9^ cells/L [14,18,30]. Significance was defined as an α-value <0.05 and SAS Enterprise Guide 7.15 (SAS Institute Inc., NC, USA) was used to conduct the analysis [31]. We conducted three analyses: In our first analysis, we assessed the prevalence of BEN at enrolment amongst HVTN 503 Black vaccine- and placebo- recipients. We compared it to the prevalence of neutropenia amongst Non-Hispanic Whites and Non-Hispanic Blacks reported in the National Health and Nutrition Examination Survey (NHANES) 2007–2008 dataset [32]. NHANES is run by the National Center for Health Statistics, a subsidiary of the Centers for Disease Control and Prevention, and is designed to assess the general health of adults and children living in the US. This was a cross-sectional survey of the civilian, non-institutionalized, US population [33]. Frequencies were determined for categorical measures and compared by neutrophil count status using Fisher’s exact test without multiple comparison testing. Medians and interquartile ranges (IQR) were determined for continuous measures and compared by neutrophil count status using the non-parametric Kruskal-Wallis test. Graphical plots were generated for neutrophil counts comparing the South African Black, US non-Hispanic White, and US non-Hispanic Black cohorts, stratified by age and sex, using box and leaf plots with jitter added to each point.

In the second analysis, baseline risk factors for HIV acquisition were determined through two stages: In the first stage, we built a regression model using the least absolute shrinkage and selection operator (LASSO) approach in which several potential variables were included as covariates. The LASSO model selected gender, age, status of BEN, body mass index (BMI), study arm, report of having a main partner, report of having a casual partner, number of partners, site, cannabis use, and sexual activity after substance use as the most critical values. BMI data were categorized according to the latest WHO BMI classification system [34]. Thereafter, we ran univariate logistic regression models and considered those with a p-value <0.1 for inclusion in the full multivariate regression model controlling for BEN. The final model was arrived at using the backward selection procedure. Results are presented as adjusted odds ratios (aOR), 95% confidence intervals (95% CI) and their p-values. Model fit was assessed by several tests including standard Pearson, standard Deviance, Osius, McCullagh, and RSS tests [35].

In the third analysis, we compared all adverse events reported throughout HVTN 503 in the placebo- and vaccine- recipients by BEN status. The frequency of system organ class (SOC) adverse events by BEN and their rates per 100-person-years are presented in Table 4 (adverse event severity of grade 1–5) and S1 Table (adverse event severity of grade 3–5) for the placebo and vaccine groups. SOC events with at least 2 Adverse events were compared using the Poisson regression model that accounted for overdispersion using a scaled deviance. Incidence of SOC was determined by dividing the count of SOC by person-years of follow-up. Person-years (py) were calculated as the difference in years between the enrolment and final visit dates. Our analyses were limited to data from 18-35-year-old males and non-pregnant females across the HVTN 503 and NHANES datasets.

## Results

**Fig 1:**
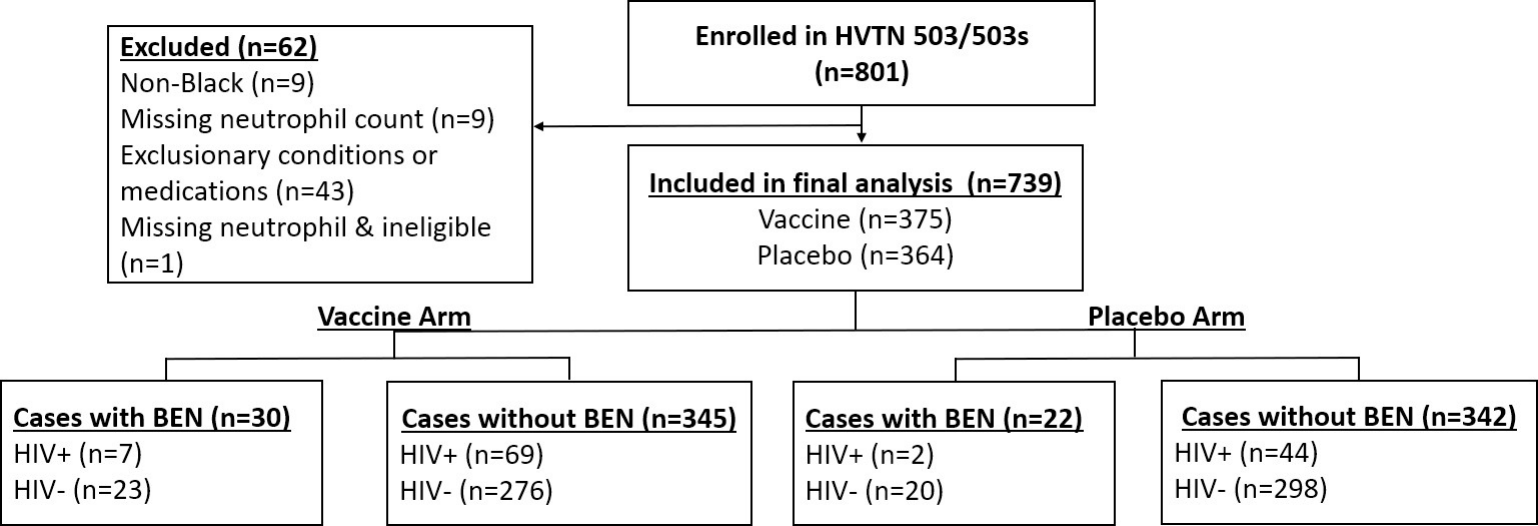
Participant disposition diagram.

The HVTN 503 dataset consisted of 801 participants that were randomized to the placebo or vaccine arms. The following participants were excluded from the analysis: 9 participants of Mixed/White ethnicity, 1 who tested HIV positive at enrolment, 9 who had missing neutrophil count measures, and 43 that were deemed ineligible due to potentially significant pre-existing conditions or concomitant medication use (E.g. respiratory tract infection, local bacterial/fungal skin infection, or vaginal discharge). Of the 43 participants excluded for a potentially significant medical condition/concomitant medication use history, 3 had neutropenia. Therefore, 739 (92%) participants were included in the analyses (Fig 1), of whom 454 participants were also enrolled in the HVTN 503-S follow-up study. Amongst the 739 participants in the South African cohort, the median age was 22 (IQR: 20–26) years; the majority were 18–24 years (n = 481, 65%) and of male gender (n = 412, 56%). The NHANES database participants matched to the HVTN 503 were 845 (540 US Non-Hispanic Whites and 305 Non-Hispanic Blacks); 375 (44%) and 470 (56%) participants were aged 18–24 and 25–34 years, respectively and their median age was 26 (IQR: 21–30). There were 457 (54%) males and 388 (46%) females. Gender proportions between the two cohorts were similar.

### Prevalence of BEN

**Fig 2:**
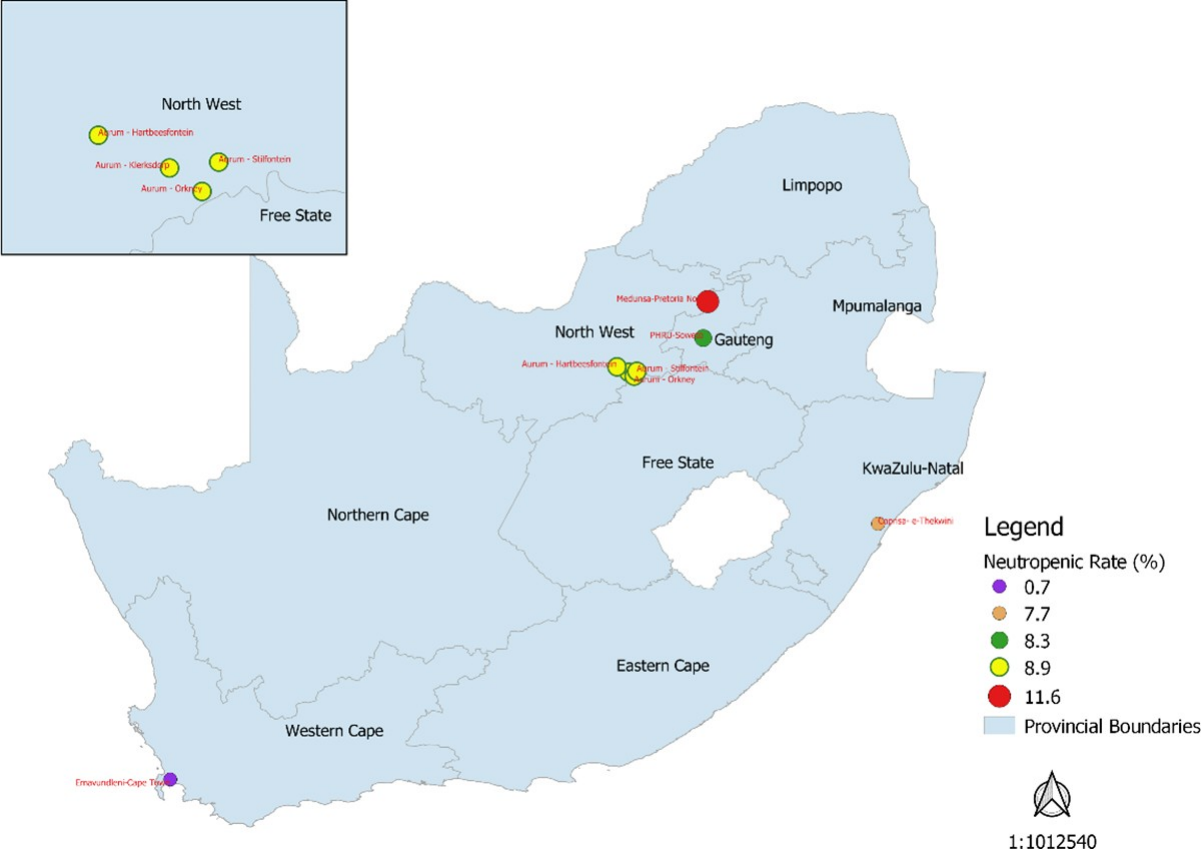
Prevalence rates of BEN in each HVTN 503 clinical research site and its representative area. Medunsa-Pretoria (red circle), PHRU-Soweto (green circle), Aurum-KOSH (yellow circles), d) Caprisa-eThekwini (orange circle), Emavundleni-Cape Town (purple circle).

The prevalence of BEN in the South African cohort was 7% (52/739). Participants with BEN had a lower median neutrophil count (1.3 vs. 3.2x10^9^ cells/L; p<0.001) and BMI (20.8 vs. 22.3 kg/m^2^; p<0.001) when compared to those without BEN, respectively (Table 1). BEN prevalence varied by geographic location (Fig 2), with Pretoria North (11.6%, 5/43) having the highest prevalence, and Cape Town having the lowest (0.7%, 1/152). Forty-seven of the 52 BEN participants had a neutrophil count between 1.0–1.5 x10^9^ cells/L (90.4%), 4 had neutrophil counts between 0.5–1.0x10^9^ cells/L (7.7%) and one participant had a neutrophil count below 0.5x10^9^ cells/L (1.9%). Approximately 38.5% (20/52) of participants with BEN reported cannabis use in the South African cohort, compared to 20.8% (143/687, p = 0.005) of participants in the non-BEN cohort.

**Table 1:** Demographic and clinical characteristics of Black participants in HVTN 503.

	Overall				Placebo				Vaccine			
Variable	Overall (n = 739)	BEN (n = 52)	Non-BEN (n = 687)	P-Value	Overall (n = 364)	BEN (n = 22)	Non-BEN (n = 342)	P-Value	Overall (n = 375)	BEN (n = 30)	Non-BEN (n = 345)	P-Value
**Age group (years)**												
18–24	481 (65.09)	36 (69.23)	445 (64.77)	0.5502	237 (65.11)	16 (72.73)	221 (64.62)	0.4978	244 (65.07)	20 (66.67)	224 (64.93)	1.0000
≥25	258 (34.91)	16 (30.77)	242 (35.23)		127 (34.89)	6 (27.27)	121 (35.38)		131 (34.93)	10 (33.33)	121 (35.07)	
Median (IQR)	22.0 (20.0–26.0)	21.0 (20.0–25.0)	22.0 (20.0–27.0)	0.1917	22.0 (20.0–26.0)	21.0 (19.0–25.0)	22.0 (20.0–27.0)	0.0448	23.0 (20.0–26.0)	22.0 (20.0–27.0)	23.0 (20.0–26.0)	0.9852
**Gender**												
Female	327 (44.25)	10 (19.23)	317 (46.14)	<0.001	163 (44.78)	3 (13.64)	160 (46.78)	0.0031	164 (43.73)	7 (23.33)	157 (45.51)	0.0210
Male	412 (55.75)	42 (80.77)	370 (53.86)		201 (55.22)	19 (86.36)	182 (53.22)		211 (56.27)	23 (76.67)	188 (54.49)	
**Treatment arm**												
Placebo	364 (49.26)	22 (42.31)	342 (49.78)	0.3170	NA	NA	NA	NA	NA	NA	NA	NA
Vaccine	375 (50.74)	30 (57.69)	345 (50.22)		NA	NA	NA	NA	NA	NA	NA	NA
**HIV Status**												
Negative	617 (83.49)	43 (82.69)	574 (83.55)	0.8470	318 (87.36)	20 (90.91)	298 (87.13)	1.0000	299 (79.73)	23 (76.67)	276 (80.00)	0.6394
Positive	122 (16.51)	9 (17.31)	113 (16.45)		46 (12.64)	2 (9.09)	44 (12.87)		76 (20.27)	7 (23.33)	69 (20.00)	
**Neutrophil count (x10**^**9**^**)**												
Median (IQR)	3.06 (2.13–4.41)	1.30 (1.17–1.43)	3.18 (2.28–4.48)	<0.001	3.16 (2.21–4.45)	1.31 (1.16–1.43)	3.27 (2.33–4.52)	<0.001	2.98 (2.07–4.36)	1.30 (1.18–1.42)	3.13 (2.25–4.44)	<0.001
**BMI (kg/m**^**2**^**)**												
Underweight	92 (12.45)	9 (17.31)	83 (12.08)	-	46 (12.64)	4 (18.18)	42 (12.28)	-	46 (12.27)	5 (16.67)	41 (11.88)	-
Normal	423 (57.24)	37 (71.15)	386 (56.19)		212 (58.24)	16 (72.73)	196 (57.31)		211 (56.27)	21 (70.00)	190 (55.07)	
Overweight	105 (14.21)	4 (7.69)	101 (14.70)		51 (14.01)	2 (9.09)	49 (14.33)		54 (14.40)	2 (6.67)	52 (15.07)	
Obese	119 (16.10)	2 (3.85)	117 (17.03)		55 (15.11)	0 (0.00)	55 (16.08)		64 (17.07)	2 (6.67)	62 (17.97)	
Median (IQR)	22.1 (19.8–26.0)	20.8 (19.4–22.1)	22.3 (19.8–26.4)	<0.001	22.0 (19.8–25.4)	20.2 (19.4–21.5)	22.2 (19.9–25.6)	0.0052	22.1 (19.7–26.7)	20.9 (19.3–22.3)	22.5 (19.8–27.0)	0.0300
Circumcision* **(males only)**												
No	472 (63.87)	27 (51.92)	445 (64.77)	0.0725	234 (64.29)	8 (36.36)	226 (66.08)	0.0098	238 (63.47)	19 (63.33)	219 (63.48)	1.0000
Yes	267 (36.13)	25 (48.08)	242 (35.23)		130 (35.71)	14 (63.64)	116 (33.92)		137 (36.53)	11 (36.67)	126 (36.52)	
**Cannabis use**												
No	576 (77.94)	32 (61.54)	544 (79.18)	0.0051	283 (77.75)	10 (45.45)	273 (79.82)	<0.001	293 (78.13)	22 (73.33)	271 (78.55)	0.4943
Yes	163 (22.06)	20 (38.46)	143 (20.82)		81 (22.25)	12 (54.55)	69 (20.18)		82 (21.87)	8 (26.67)	74 (21.45)	
**Heavy drinking**												
No	624 (84.55)	45 (86.54)	579 (84.40)	0.8427	302 (83.20)	18 (81.82)	284 (83.28)	0.7736	322 (85.87)	27 (90.00)	295 (85.51)	0.7837
Yes	114 (15.45)	7 (13.46)	107 (15.60)		61 (16.80)	4 (18.18)	57 (16.72)		53 (14.13)	3 (10.00)	50 (14.49)	
**Site**												
Cape Town	152 (20.57)	1 (1.92)	151 (21.98)	-	74 (20.33)	0 (0.00)	74 (21.64)	-	78 (20.80)	1 (3.33)	77 (22.32)	-
Caprisa	52 (7.04)	4 (7.69)	48 (6.99)		27 (7.42)	1 (4.55)	26 (7.60)		25 (6.67)	3 (10.00)	22 (6.38)	
KOSH	202 (27.33)	18 (34.62)	184 (26.78)		99 (27.20)	9 (40.91)	90 (26.32)		103 (27.47)	9 (30.00)	94 (27.25)	
Medunsa	43 (5.82)	5 (9.62)	38 (5.53)		20 (5.49)	2 (9.09)	18 (5.26)		23 (6.13)	3 (10.00)	20 (5.80)	
Soweto-PHRU	290 (39.24)	24 (46.15)	266 (38.72)		144 (39.56)	10 (45.45)	134 (39.18)		146 (38.93)	14 (46.67)	132 (38.26)	

**NB:** BEN prevalence is 8.3% in Soweto, 0.7% in Cape Town, 8.9% in KOSH, 7.7% in Caprisa and 11.6% in Medunsa

*Circumcision rates affect only males; Categorical values comparing the proportion of participants with and without BEN were compared by Fishers Exact test analysis without multiple comparison testing; Median values were compared by the Kruskal-Wallis test.

### Population comparison of Neutrophil counts

**Fig 3:**
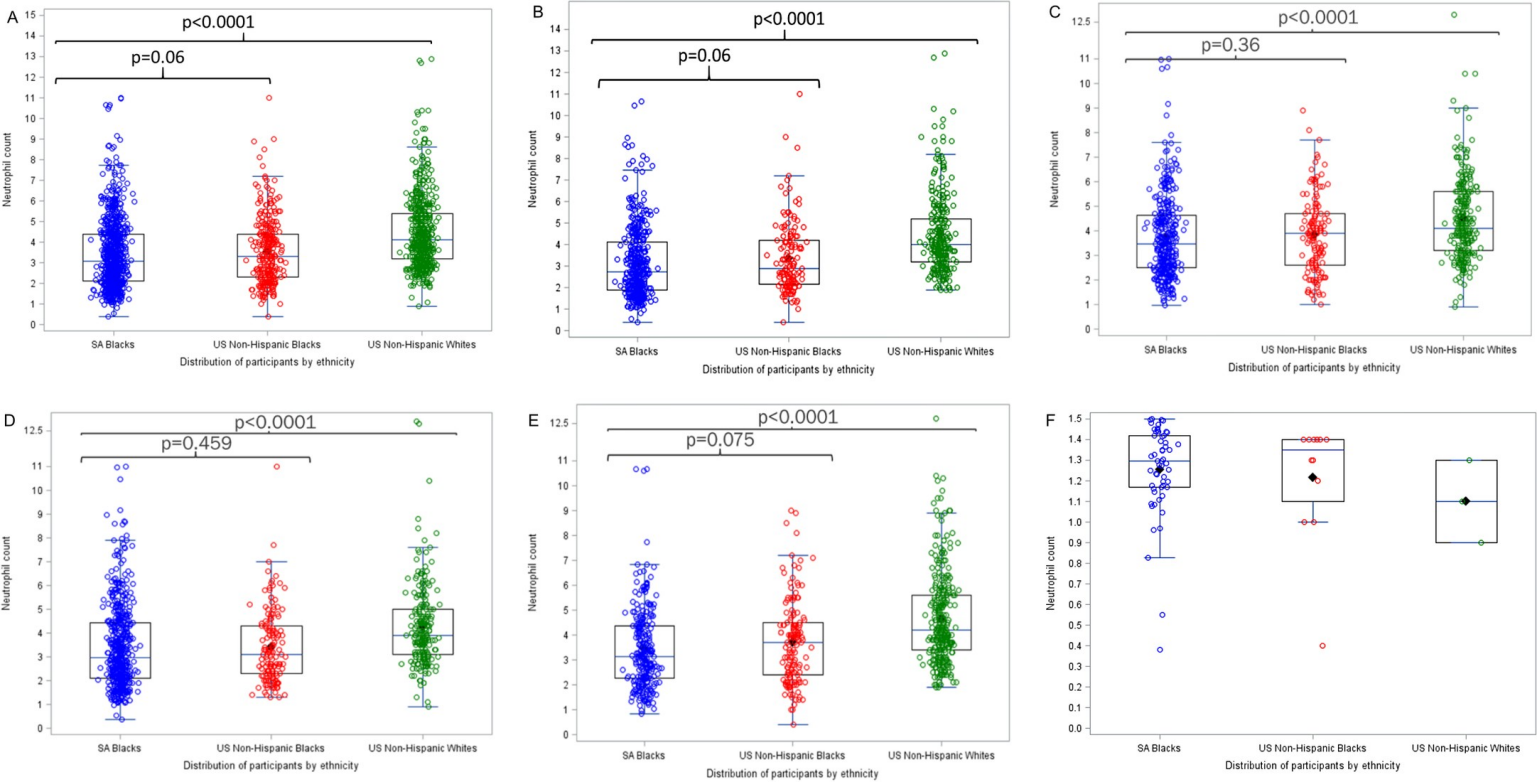
Neutrophil counts between the South African and US cohorts by ethnicity. 3A) Neutrophil counts by ethnicity. 3B) Neutrophil counts among males, 3C) Neutrophil counts among females, 3D) Neutrophil counts among 18-24-year-olds, 3E) Neutrophil counts among ≥25-year-olds, 3F) Neutrophil counts among those with BEN.

In the comparison between the South African cohort (n = 739) and the NHANES comparator group comprising of US non-Hispanic Whites (n = 540), the median neutrophil count in the Black South African cohort was lower than the non-Hispanic White population (3.06 [IQR: 2.13–4.40] vs. 4.1 [IQR: 3.2–5.4]; p<0.001). The proportion of participants with neutropenia was significantly higher in South African Blacks than in Non-Hispanic Whites (7% [52/739] vs. 0.6% [3/540]; p<0.001). A similar trend was also noted between the South African Black and US Non-Hispanic Black cohorts, however, this difference was not significant (7% [52/739] vs. 4% [12/305]; p = 0.0648). Neutrophil count distributions stratified by ethnicity, gender, and age are illustrated in Fig 3.

Among the HVTN 503 laboratory measures assessed at enrolment (Table 2), participants with BEN had significantly lower median CD4 (663 vs. 771 cells/mm^3^; p = 0.0044), leucocyte (3.4 vs. 5.7x10^9^ cells/L; p<0.001), platelet (252 vs. 281x10^9^ cells/L; p = 0.0058), monocyte (0.25 vs. 0.35x10^9^ cells/L; p<0.001) and basophil (0.02 vs. 0.03x10^9^ cells/L; p = 0.0236) counts compared to those without BEN. Similarly, the neutrophil-lymphocyte ratio (0.8 vs. 1.7; p<0.001) was significantly lower in participants with BEN. References for the laboratory measures are also presented in Table 2 [36–38].

**Table 2:** Median (IQR) laboratory measures at baseline by BEN of Black participants in HVTN 503.

Variable	Overall				Placebo				Vaccine				Reference ranges
	Overall	BEN	Non-BEN	p-value	Overall	BEN	Non-BEN	p-value	Overall	BEN	Non-BEN	p-value	
**CD4 Count (/mm**^**3**^**)**	762.0 (608.0–938.0)	663.0 (533.5–844.0)	771.0 (612.0–941.0)	0.0044	776.5 (610.5–952.0)	703.0 (536.0–941.0)	781.0 (613.0–958.0)	0.2270	750.0 (600.0–924.0)	634.5 (517.0–763.0)	760.0 (612.0–935.0)	0.0079	600–1500
**CD8 Count (/mm**^**3**^**)**	489.0 (375.0–630.0)	455.0 (348.0–604.5)	491.0 (380.0–632.0)	0.2278	492.0 (375.0–639.0)	433.5 (314.0–567.0)	493.5 (386.0–642.0)	0.0888	484.0 (374.0–610.0)	469.5 (352.0–620.0)	485.0 (378.0–607.0)	0.9126	400–1000
**White Blood Cell (x10**^**9**^**/L)**	5.5 (4.4–6.9)	3.4 (3.0–3.75)	5.7 (4.6–7.1)	<0.001	5.7 (4.4–7.1)	3.3 (2.9–3.9)	5.8 (4.6–7.1)	<0.001	5.3 (4.3–6.8)	3.4 (3.0–3.7)	5.5 (4.6–6.9)	<0.001	4.0–12.0
**Hemoglobin (g/dl)**	14.8 (13.4–15.9)	15.4 (14.4–16.1)	14.7 (13.4–15.9)	0.0255	14.8 (13.35–15.8)	15.65 (15.1–16.10)	14.7 (13.3–15.8)	0.0224	14.8 (13.5–16.0)	15.3 (14.2–16.1)	14.7 (13.5–16.0)	0.3288	13.8–18.8
**Hematocrit (%)**	44.0 (40.0–47.0)	46.0 (43.5–47.5)	44.0 (39.9–47.0)	0.0105	44.0 (39.8–47.0)	46.5 (45.0–48.0)	44.0 (39.4–47.0)	0.0119	44.0 (40.4–47.0)	45.0 (43.0–47.0)	44.0 (40.0–47.0)	0.2274	Male: 0.40–0.56 Female: 0.35–0.49
**Mean Corpuscular Volume (fl)**	90.3 (86.4–93.3)	90.6 (86.2–93.6)	90.3 (86.4–93.3)	0.6212	89.9 (86.5–93.1)	90.6 (88.4–92.8)	89.9 (86.2–93.1)	0.7168	90.7 (86.3–93.6)	90.7 (84.1–94.7)	90.6 (86.5–93.4)	0.7019	79.0–100.0
**Platelets (x10**^**9**^**/L)**	278.0 (239.0–328.0)	252.0 (211.0–311.0)	281.0 (241.5–330.0)	0.0058	283.0 (244.0–335.0)	271.0 (212.0–316.0)	284.0(245.0–337.5)	0.2235	275.5 (238.0–323.0)	244.0 (206.0–310.0)	277.5 (239.0–324.0)	0.0118	150–450
**Neutrophils (x10**^**9**^**)**	3.06 (2.13–4.41)	1.30 (1.17–1.43)	3.19 (2.28–4.48)	<0.001	3.16 (2.21–4.45)	1.31 (1.16–1.44)	3.27 (2.33–4.52)	<0.001	2.98 (2.07–4.36)	1.30 (1.18–1.42)	3.13 (2.25–4.44)	<0.001	2.0–7.5
**Lymphocytes(x10**^**9**^**/L)**													
Median (IQR)	1.80 (1.47–2.21)	1.63 (1.44–2.03)	1.82 (1.47–2.24)	0.0654	1.83 (1.46–2.29)	1.57 (1.43–1.99)	1.85 (1.47–2.30)	0.`0	1.78 (1.47–2.13)	1.65 (1.44–2.07)	1.79 (1.47–2.14)	0.3663	1.0–4.0
**Neutrophil/Lymphocytes ratio**	1.69 (1.15–2.42)	0.78 (0.62–0.92)	1.74 (1.23–2.50)	<0.001	1.70 (1.11–2.41)	0.77 (0.63–0.90)	1.77 (1.23–2.46)	<0.001	1.64 (1.17–2.43)	0.78 (0.58–0.93)	1.74 (1.24–2.51)	<0.001	1–3
**Monocytes (x10**^**9**^**/L)**	0.35 (0.28–0.44)	0.25 (0.215–0.30)	0.35 (0.29–0.45)	<0.001	0.35 (0.28–0.46)	0.24 (0.21–0.30)	0.36 (0.29–0.46)	<0.001	0.34 (0.28–0.43)	0.27 (0.21–0.32)	0.35 (0.29–0.44)	<0.001	0.2–1.0
**Eosinophils (x10**^**9**^**/L)**	0.09 (0.05–0.17)	0.07 (0.04–0.14)	0.09(0.05–0.17)	0.1263	0.10 (0.05–0.17)	0.10 (0.04–0.15)	0.09 (0.05–0.17)	0.5163	0.08 (0.05–0.15)	0.07 (0.04–0.13)	0.09 (0.05–0.17)	0.1638	0–0.5
**Basophils (x10**^**9**^**/L)**	0.03 (0.02–0.05)	0.02 (0.02–0.04)	0.03 (0.02–0.05)	0.0236	0.03(0.02–0.05)	0.02 (0.02–0.04)	0.03 (0.02–0.05)	0.2093	0.03 (0.02–0.05)	0.02 (0.01–0.04)	0.03 (0.02–0.05)	0.0517	0–0.3

**NB:** Median values were compared by the Kruskal-Wallis test.

### HIV acquisition and BEN

In the placebo group, 364 participants were followed up for a total of 1662.0 py (124.1 for BEN and 1537.96 for those without BEN). Of those, 12.6% (46/364) acquired HIV (2 in the BEN group and 44 in those without BEN). Of the 2 participants that acquired HIV in the BEN group, 1/163 (0.6%) was female and 1/201 (0.5%) was male; they acquired HIV in the second and 7^th^ year of follow-up, respectively. In the vaccine group, 20% (76/375) of the participants had acquired HIV after 1686.5 years of follow-up (126.9 for BEN and 1559.6 for those without BEN). Of the 76 participants, 7 (9.2%) had BEN and 69 (90.8%) did not have BEN. Among the HIV-infected, 40/76 (52.6%) were female and 36/76 male (47.4%). Additionally, 9.2% (7/76) of these participants in the vaccine group had BEN, while 69/76 (90.8%) did not have BEN. Five of those with BEN acquired HIV at the 1st, 2nd, 4^th^, 6^th,^ and 7^th^ year of follow-up. The remaining 2 acquired HIV in the 3^rd^ year of follow-up. Analysis by gender showed that 4/164 (2.4%) females with BEN acquired HIV in the vaccine group, compared to 3/211 (1.4%) males. No association was observed between HIV acquisition and BEN in the placebo (p = 1.00, Table 1) or vaccine groups (p = 0.84, Table 1).

In the multivariate logistic regression assessing the baseline risk factors for HIV acquisition (Table 3), there was no significant difference by the status of BEN for the odds of HIV infection (aOR: 1.364, 95% CI: 0.625–2.976; p = 0.4351). However, female gender (aOR: 1.947, 95% CI: 1.265–2.996; p = 0.0025) and cannabis use (aOR: 2.192, 95% CI: 1.126–4.266; p = 0.0209) increased the odds of HIV acquisition. Those enrolled in the placebo arm (aOR: 0.569, 95% CI: 0.382–0.848; p = 0.0051) had lower odds of HIV infection. All model fit statistic tests used to assess the model fit returned non-significant p-values confirming a good fit.

**Table 3:** Baseline predictors of HIV infection among HVTN 503 black participants.

		Univariate		Multivariate	
Variable	N or Median (IQR)	OR (95%CI)	p-value	OR (95%CI)	p-value
**Gender**					
Female	327 (44%)	2.311 (1.552–3.443)	<0.001	1.947 (1.265–2.996)	**0.0025**
Male	412 (56%)	Ref		Ref	
**Age (years)**	20 (IQR: 22–26)	1.034 (0.991–1.079)	0.1224		
**BEN Status**					
BEN	52 (7%)	1.063 (0.504–2.242)	0.8722	1.364 (0.625–2.976)	0.4351
No BEN	687 (93%)	Ref		Ref	
**BMI**	19.8 (IQR: 22.1–26.0)	1.026 (0.998–1.055)	0.0722		
**Study Arm**					
Placebo	364 (49%)	0.569 (0.382–0.848)	0.0056	0.560 (0.373–0.840)	**0.0051**
Vaccine	375 (51%)	Ref		Ref	
**Participant reports having a main partner**					
Yes	560 (76%)	1.456 (0.893–2.374)	0.1317		
No or Didn't have	179 (24%)	Ref			
**Number of partners**	1 (IQR: 1–2)	0.847 (0.706–1.015)	0.0721		
**Site**					
Cape Town	152 (21%)	1.62 (0.971–2.700)	0.0645		
Caprisa	52 (7%)	2.950 (1.514–5.747)	0.0015		
KOSH	202 (27%)	0.897 (0.529–1.521)	0.6871		
Medunsa	43 (6%)	0.985 (0.391–2.480)	0.9741		
Soweto	290 (39%)	Ref			
**Participant reports having a casual partner**					
Yes	230 (31%)	0.548 (0.345–0.873)			
No or Didn't have	509 (69%)	Ref			
**Sex after Alcohol/Drug use**					
Yes	199 (27%)	0.777 (0.492–1.227)	0.2794		
No	540 (73%)	Ref			
**Cannabis use**					
Yes	163 (22%)	0.337 (0.181–0.628)	0.006	2.192 (1.126–4.266)	**0.0209**
No	576 (78%)	Ref			

**NB:** REF is the reference category.

### Adverse events and BEN

Most adverse events (Grades 1–5, Table 4) occurred in the infections and infestations SOC, followed by the metabolism and nutrition disorders SOC when adverse events of all severities were analysed (Grades 1–5). In the placebo arm, 12.1 (95% CI: 7.3–20.1) events per 100py in the infections and infestations SOC were noted among participants with BEN compared to 16.5 (95% CI: 14.6–18.7) events per 100py in the non-BEN cohort (p = 0.0625). The vaccine treatment group with BEN had an event incidence rate of 19.7 (95% CI: 13.3–29.2) per 100py, compared to 14.8 (95% CI: 13.0–16.8) events per 100py in the group without BEN (p = 0.07). An assessment of severe adverse events (grade 3–5) showed that most of them occurred in the metabolism and nutritional disorders SOC (S1 Table).

**Table 4:** Adverse events (Grade 1–5) by system organ class among Black participants in HVTN 503.

	Placebo						Vaccine					
	BEN			Non-BEN			BEN			Non-BEN		
SOC/PT	n (%)	Events	IR (95% CI)	n (%)	Events	IR (95% CI)	n (%)	Events	IR (95% CI)	n (%)	Events	IR (95% CI)
**Infections and infestations**	8 (36.4)	15	12.1 (7.3,20.1)	158 (46.2)	253	16.5 (14.6,18.7)	15 (50.0)	25	19.7 (13.3,29.2)	147 (42.6)	231	14.8 (13.0,16.8)
**Metabolism and nutrition disorders**	4 (18.2)	6	4.8 (2.2,10.7)	61 (17.8)	79	5.1 (4.1,6.4)	3 (10.0)	3	2.4 (0.8,7.4)	64 (18.6)	84	5.4 (4.4,6.7)
**Blood and lymphatic system disorders**	5 (22.7)	5	4.0 (1.7,9.6)	30 (8.8)	35	2.3 (1.7,3.2)	8 (26.7)	8	6.3 (3.2,12.6)	38 (11.0)	52	3.3 (2.5,4.3)
**Reproductive system and breast disorders**	0 (0.0)	0	0.0 (0.0,0.0)	24 (7.0)	33	2.1 (1.5,3.0)	1 (3.3)	1	0.8 (0.1,5.7)	31 (9.0)	40	2.6 (1.9,3.5)
**Injury, poisoning, and procedural complications**	1 (4.5)	1	0.8 (0.1,5.7)	33 (9.6)	36	2.3 (1.7,3.2)	3 (10.0)	3	2.4 (0.8,7.4)	24 (7.0)	30	1.9 (1.3,2.7)
**Investigations**	3 (13.6)	3	2.4 (0.8,7.4)	23 (6.7)	25	1.6 (1.1,2.4)	2 (6.7)	2	1.6 (0.4,6.4)	34 (9.9)	38	2.4 (1.7,3.3)
**Nervous system disorders**	0 (0.0)	0	0.0 (0.0,0.0)	18 (5.3)	19	1.2 (0.8,1.9)	3 (10.0)	3	2.4 (0.8,7.4)	33 (9.6)	38	2.4 (1.7,3.3)
**Vascular disorders**	1 (4.5)	3	2.4 (0.8,7.4)	24 (7.0)	26	1.7 (1.2,2.5)	2 (6.7)	4	3.2 (1.2,8.5)	22 (6.4)	26	1.7 (1.2,2.5)
**Gastrointestinal disorders**	1 (4.5)	2	1.6 (0.4,6.4)	17 (5.0)	19	1.2 (0.8,1.9)	5 (16.7)	6	4.7 (2.1,10.5)	18 (5.2)	20	1.3 (0.8,2.0)
**Skin and subcutaneous tissue disorders**	0 (0.0)	0	0.0 (0.0,0.0)	17 (5.0)	19	1.2 (0.8,1.9)	2 (6.7)	2	1.6 (0.4,6.4)	19 (5.5)	22	1.4 (0.9,2.1)
**Musculoskeletal and connective tissue disorders**	2 (9.1)	2	1.6 (0.4,6.4)	11 (3.2)	11	0.7 (0.4,1.3)	1 (3.3)	1	0.8 (0.1,5.7)	16 (4.6)	18	1.2 (0.8,1.9)
**Respiratory, thoracic, and mediastinal disorders**	1 (4.5)	1	0.8 (0.1,5.7)	13 (3.8)	13	0.8 (0.5,1.4)	2 (6.7)	2	1.6 (0.4,6.4)	9 (2.6)	9	0.6 (0.3,1.2)
**General disorders and administration site conditions**	0 (0.0)	0	0.0 (0.0,0.0)	8 (2.3)	8	0.5 (0.3,1.0)	2 (6.7)	2	1.6 (0.4,6.4)	13 (3.8)	14	0.9 (0.5,1.5)
**Pregnancy, puerperium, and perinatal conditions**	0 (0.0)	0	0.0 (0.0,0.0)	13 (3.8)	16	1.0 (0.6,1.6)	0 (0.0)	0	0.0 (0.0,0.0)	7 (2.0)	8	0.5 (0.3,1.0)
**Renal and urinary disorders**	0 (0.0)	0	0.0 (0.0,0.0)	7 (2.0)	7	0.5 (0.2,1.0)	3 (10.0)	3	2.4 (0.8,7.4)	5 (1.4)	5	0.3 (0.1,0.7)
**Psychiatric disorders**	0 (0.0)	0	0.0 (0.0,0.0)	3 (0.9)	3	0.2 (0.1,0.6)	0 (0.0)	0	0.0 (0.0,0.0)	6 (1.7)	8	0.5 (0.3,1.0)
**Neoplasms benign, malignant, and unspecified (incl. cysts and polyps)**	0 (0.0)	0	0.0 (0.0,0.0)	3 (0.9)	3	0.2 (0.1,0.6)	2 (6.7)	2	1.6 (0.4,6.4)	4 (1.2)	4	0.3 (0.1,0.8)
**Eye disorders**	0 (0.0)	0	0.0 (0.0,0.0)	2 (0.6)	2	0.1 (0.0,0.4)	1 (3.3)	1	0.8 (0.1,5.7)	3 (0.9)	3	0.2 (0.1,0.6)
**Surgical and medical procedures**	0 (0.0)	0	0.0 (0.0,0.0)	3 (0.9)	3	0.2 (0.1,0.6)	0 (0.0)	0	0.0 (0.0,0.0)	2 (0.6)	2	0.1 (0.0,0.4)
**Congenital, familial, and genetic disorders**	0 (0.0)	0	0.0 (0.0,0.0)	2 (0.6)	3	0.2 (0.1,0.6)	0 (0.0)	0	0.0 (0.0,0.0)	1 (0.3)	1	0.1 (0.0,0.7)
**Cardiac disorders**	N/A	N/A	N/A	N/A	N/A	N/A	1 (3.3)	1	0.8 (0.1,5.7)	2 (0.6)	2	0.1 (0.0,0.4)
**Immune system disorders**	0 (0.0)	0	0.0 (0.0,0.0)	2 (0.6)	2	0.1 (0.0,0.4)	N/A	N/A	N/A	N/A	N/A	N/A
**Hepatobiliary disorders**	N/A	N/A	N/A	N/A	N/A	N/A	0 (0.0)	0	0.0 (0.0,0.0)	1 (0.3)	1	0.1 (0.0,0.7)

n = Number of participants experiencing an event.

% = Percentage of participants experiencing an event.

Events = Number of adverse events.

IR = Incidence rate per 100 person-years.

95% CI = 95% confidence interval of IR.

## Discussion

Our analysis found that BEN occurred in 7% of the Black South African population enrolled in HVTN 503. These participants also had lower median ANC when compared to a matched sample of Non-Hispanic Whites drawn from the general population of the US. Additionally, we were unable to demonstrate an association between BEN and HIV acquisition. However, female gender, cannabis use, and randomization to the HVTN 503 vaccine treatment arm were associated with an increased risk for HIV acquisition. Finally, no association was found between BEN and the incidence of adverse events, including infections and infestations.

BEN has been described in research conducted in populations of African, Middle Eastern and Caribbean ancestry [19,21,30]. However, few studies have been performed within the South African population. Literature has reported BEN prevalence rates of up to 50% in certain populations [14–21], but comparing results from these studies is challenging as various cut-off reference values have been used. Our study found an overall prevalence of 7% when a lower reference limit of 1.5x10^9^ cells/L was used to define neutropenia. The establishment of ANC reference ranges based on racial or ethnic differentiation has been considered by numerous authors previously [12–14]. Implementation of ethnicity-specific reference ranges for ANC might prove logistically challenging but may serve as a solution to issues encountered by the use of one reference range for various ethnicities, which may negatively affect patient management, e.g. deciding when to initiate or stop chemotherapy based on neutrophil values, or the inappropriate use of antibiotics in conditions based on a diagnosis of presumed sepsis/bacteraemia due to a benign, decreased neutrophil count [9,39,40].

Most participants with neutropenia in our analysis had ANC between 1.0–1.5x10^9^ cells/L. However, this reduction was not significantly associated with an increased incidence of infections. It is well established that leucocytes, particularly neutrophils, are vital in providing antimicrobial protection against bacteria and fungi. In individuals with acute leukaemia, the risk of severe infection was inversely proportional to the leucocyte count [41]. In addition, the risk of severe infections is greatly increased when the neutrophil count falls below 0.5 x10^9^ cells/L [42]. It has been suggested that BEN is caused by neutrophil marginalisation in the bone marrow resulting in reduced circulating neutrophils, rather than a reduction in the total number of systemic neutrophils [14,19]. The hypothesis that BEN does not result in impaired neutrophil-mediated immunity is supported by our findings, wherein we failed to find an association between reported adverse events and BEN, particularly adverse events of an infectious aetiology. A previous study that analysed gene expression levels in an African-American cohort was also unable to find a significant difference between participants with BEN and those with normal neutrophil counts, suggesting that circulating neutrophils from individuals with BEN are functionally similar to those without BEN [43]. To our knowledge, this is the first study that sought to investigate the association between BEN and the frequency or type of adverse events in a clinical trial of healthy adults. These results are reassuring that the presence of BEN is unlikely to have a significant effect on adverse event reporting in HIV vaccine clinical trials.

A large amount of variability was noted in the distribution of BEN among the various South African regions sampled. Participants from the Pretoria North region were noted to have the highest prevalence of neutropenia when compared with other regions. Interestingly, Black participants in Cape Town had a much lower prevalence of neutropenia (0.7%) when compared with Black participants from other regions. Recent literature has strongly implicated a genetic aetiology for BEN. More specifically, *rs2814778*, a single nucleotide polymorphism (SNP) on chromosome 1, was the strongest predictor of neutropenia in 4 recent studies that conducted genome analyses to investigate possible genetic marker associations with BEN. This SNP has also been linked with a Duffy null phenotype associated with the lack of Duffy Antigen Receptor for Chemokine (DARC) expression on erythrocyte cell membranes [16,44–46]. A visual analysis of the geographical variation of BEN in our South African cohort demonstrated a general decline in the prevalence of BEN in comparison to the northern region of South Africa, with Cape Town located in the southern region. A similar pattern has been reported in epidemiological studies that showed steep “clines” in the DARC null phenotype from the Central African belt which demonstrated prevalence rates ≥90%, to the southernmost point in Africa which had median frequencies of <10% [47,48]. Considering that this marker appears to be a strong indicator of African ancestry in the majority of regions in Sub-Saharan Africa, it is worth hypothesizing that the reduced prevalence of neutropenia in the Cape Town region may be indicative of genetic admixture. Alternatively, this observation may, at least in part, be due to a historically decreased prevalence of malaria due to *Plasmodium vivax* in the southern African region [16,48,49]. This is relevant as the currently accepted aetiology for BEN is that the DARC-null phenotype conferred an evolutionary advantage to infection by *P*. *vivax*, which allowed for genetic fixation in Sub-Saharan Africa [47,50,51]. Populations residing in areas with decreased *P*. *vivax* infection will, therefore, have been less likely to positively select the genetic SNP, thereby resulting in a decreased prevalence of BEN.

Research on contributory factors for BEN have previously included genetic and environmental factors, and the aetiology is likely multifactorial. The findings in this paper support this, as cannabis use and BMI were significantly associated with the prevalence of BEN. Previous murine and *in-vitro* data have shown that cannabinoid compounds inhibit neutrophil migration and function, which may have negative effects on the neutrophil cell count [52,53]. However, data on associations between cannabis use and BEN are scarce. This may have important consequences for patient care in therapeutic areas such as psychiatry where cannabis use is frequent and likely to become more prevalent in the current context of cannabis legalisation in South Africa [54]. Additionally, neutropenia in a psychotic patient with concomitant clozapine and cannabis use may, therefore, be incorrectly attributed to clozapine, instead of cannabis. The concomitant use of these agents may also exert additive myelosuppressive effects that further increase the patient’s risk for agranulocytosis [40,55] Further research is warranted to investigate these associations. A significant difference in BMI between participants with BEN and those with normal neutrophil counts also correlates with data that elevated neutrophil counts were associated with an increased BMI in adolescents with asthma [56]. The aetiology for this association is unclear but may either relate to an altered inflammatory state in individuals with an increased BMI, thus resulting in increased neutrophil counts, or it may be due to linkage disequilibrium between genetic polymorphisms that contribute towards an increased BMI and SNPs associated with BEN [57].

The results from our study showed that, at a lower limit of 1.5 x10^9^ cells/L, the presence of BEN did not significantly influence the participants’ susceptibility for HIV infection. Previous research is conflicting, with some studies demonstrating an increased susceptibility for HIV infection with decreased neutrophil counts [4,6], while others failed to show an association [58–60]. While the role of the adaptive immune system in the pathogenesis of HIV has been well described [61], the innate immune system’s role is less clear. Neutrophils are among the first cell lines to reach the site of infection, and several mechanisms have been proposed and described to contribute to its protective action against HIV, including phagocytosis of HIV, neutrophil extracellular trap formation, and chemokine modulation [62,63]. Various reasons may account for the contrast in results, including differences in the risk profile of the sampled population, tribe specific characteristics, or differing prevalence rates of BEN based on the reference ranges used [9]. Overall, these results provide reassurance that the inclusion of participants with BEN in HIV vaccine clinical trials is unlikely to place them at additional risk for HIV infection.

On the contrary, female individuals and those that reported cannabis use had an approximately 2-fold increase in their risk for HIV infection. While it is well described that female individuals are at a disproportionately increased risk for HIV acquisition [64], the evidence around cannabis use as a risk factor is conflicting. A study by Woody et al was unable to demonstrate an association between cannabis use and higher sexual risk [65], but a paper published in 2015 reported that marijuana use among Black men who have sex with men was associated with a significantly increased risk for HIV acquisition [66]. Similarly, another study reported that young, heterosexual adults that used cannabis had a higher risk preference, decreased intention to use HIV protective measures, and lower HIV self-efficacy—factors that increase their HIV acquisition risk [67]. It is critical that methodologies such as HIV risk reduction counselling that are performed in most HIV vaccine clinical trials should pay particular attention to the frequent use of cannabis, and explore these risks with their participants using a non-judgmental and open-minded approach.

This study had some limitations. Our data were collected from 4 of the 9 provinces in South Africa, and thus, may not be a completely representative sample of the South African population. Our analyses were limited to complete blood count data collected at enrolment, and these data, therefore, do not account for natural fluctuations in ANC. Additionally, the NHANES dataset utilized a different study design and sampling approach, which may have introduced bias, however, this was mitigated using a time- and age-matched sample from the dataset. The ethnic categorization used in the NHANES dataset also differed from the self-designated racial categories used in the HVTN 503 study, which may have influenced our results. Lastly, HVTN 503 was not powered to fully assess the effect of BEN on HIV acquisition or adverse event reporting.

## Conclusions

BEN is common in South Africa but there is significant geographical variation, providing future opportunities for genetic research to elucidate differences in HIV susceptibility and adverse events. Furthermore, our findings highlight the importance of prevention strategies continuing to focus on female gender and cannabis use, which were statistically significant risk factors for HIV acquisition in South Africa. This is especially relevant in the current context of cannabis legalisation in South Africa. Finally, our findings provide reassurance that the inclusion of BEN participants in HIV vaccine clinical trials is unlikely to present additional safety issues.

## Supporting information

S1 Table Adverse events (Grade 3–5) by system organ class among Black participants in HVTN 503. (DOCX)

## Abbreviations

ANC: Absolute neutrophil count
aOR: adjusted odds ratio
BEN: Benign ethnic neutropenia
BMI: body mass index
CI: confidence interval
DARC: Duffy antigen receptor for chemokines
IQR: Interquartile range
NHANES: National health and nutrition examination survey
py: Person-years
SNP: single nucleotide polymorphism
SOC: System organ class
US: United States
