# Supplementary material for: Benign ethnic neutropenia in a South African population, and its association with HIV acquisition and adverse event reporting in an HIV vaccine clinical trial
Source: PLoS One. 2021 Jan 22;16(1):e0241708. doi: 10.1371/journal.pone.0241708 (PMC7822320; doi:10.1371/journal.pone.0241708)
Supplement: S1 Table — (DOCX) [file pone.0241708.s001.docx]

**S1 Table: Adverse events (Grade 3-5) by system organ class among black participants in HVTN 503**

|  | **Placebo** | | | | | | **Vaccine** | | | | | |
| --- | --- | --- | --- | --- | --- | --- | --- | --- | --- | --- | --- | --- |
|  | **BEN** | | | **Non-BEN** | | | **BEN** | | | **Non-BEN** | | |
| **SOC/PT** | **n (%)** | **Events** | **IR (95% CI)** | **n (%)** | **Events** | **IR (95% CI)** | **n (%)** | **Events** | **IR (95% CI)** | **n (%)** | **Events** | **IR (95% CI)** |
| **Metabolism and nutrition disorders** | 2 (9.1) | 2 | 1.6 (0.4,6.4) | 19 (5.6) | 22 | 1.4 (0.9,2.1) | 0 (0.0) | 0 | 0.0 (0.0,0.0) | 21 (6.1) | 26 | 1.7 (1.2,2.5) |
| **Injury, poisoning, and procedural complications** | 1 (4.5) | 1 | 0.8 (0.1,5.7) | 11 (3.2) | 11 | 0.7 (0.4,1.3) | 2 (6.7) | 2 | 1.6 (0.4,6.4) | 8 (2.3) | 8 | 0.5 (0.3,1.0) |
| **Infections and infestations** | 0 (0.0) | 0 | 0.0 (0.0,0.0) | 6 (1.8) | 11 | 0.7 (0.4,1.3) | 0 (0.0) | 0 | 0.0 (0.0,0.0) | 5 (1.4) | 6 | 0.4 (0.2,0.9) |
| **Pregnancy, puerperium, and perinatal conditions** | 0 (0.0) | 0 | 0.0 (0.0,0.0) | 8 (2.3) | 9 | 0.6 (0.3,1.2) | 0 (0.0) | 0 | 0.0 (0.0,0.0) | 6 (1.7) | 7 | 0.4 (0.2,0.8) |
| **Nervous system disorders** | 0 (0.0) | 0 | 0.0 (0.0,0.0) | 1 (0.3) | 1 | 0.1 (0.0,0.7) | 1 (3.3) | 1 | 0.8 (0.1,5.7) | 4 (1.2) | 5 | 0.3 (0.1,0.7) |
| **Investigations** | 0 (0.0) | 0 | 0.0 (0.0,0.0) | 1 (0.3) | 1 | 0.1 (0.0,0.7) | 2 (6.7) | 2 | 1.6 (0.4,6.4) | 3 (0.9) | 3 | 0.2 (0.1,0.6) |
| **Vascular disorders** | 0 (0.0) | 0 | 0.0 (0.0,0.0) | 4 (1.2) | 4 | 0.3 (0.1,0.8) | 0 (0.0) | 0 | 0.0 (0.0,0.0) | 2 (0.6) | 2 | 0.1 (0.0,0.4) |
| **Blood and lymphatic system disorders** | 0 (0.0) | 0 | 0.0 (0.0,0.0) | 1 (0.3) | 1 | 0.1 (0.0,0.7) | 0 (0.0) | 0 | 0.0 (0.0,0.0) | 4 (1.2) | 4 | 0.3 (0.1,0.8) |
| **Psychiatric disorders** | 0 (0.0) | 0 | 0.0 (0.0,0.0) | 1 (0.3) | 1 | 0.1 (0.0,0.7) | 0 (0.0) | 0 | 0.0 (0.0,0.0) | 2 (0.6) | 4 | 0.3 (0.1,0.8) |
| **Congenital, familial, and genetic disorders** | 0 (0.0) | 0 | 0.0 (0.0,0.0) | 1 (0.3) | 2 | 0.1 (0.0,0.4) | 0 (0.0) | 0 | 0.0 (0.0,0.0) | 1 (0.3) | 1 | 0.1 (0.0,0.7) |
| **Gastrointestinal disorders** |  |  |  |  |  |  | 0 (0.0) | 0 | 0.0 (0.0,0.0) | 3 (0.9) | 3 | 0.2 (0.1,0.6) |
| **General disorders and administration site conditions** | 0 (0.0) | 0 | 0.0 (0.0,0.0) | 1 (0.3) | 1 | 0.1 (0.0,0.7) |  |  |  |  |  |  |
| **Hepatobiliary disorders** | N/A | N/A | N/A | N/A | N/A | N/A | 0 (0.0) | 0 | 0.0 (0.0,0.0) | 1 (0.3) | 1 | 0.1 (0.0,0.7) |
| **Musculoskeletal and connective tissue disorders** | 0 (0.0) | 0 | 0.0 (0.0,0.0) | 1 (0.3) | 1 | 0.1 (0.0,0.7) | N/A | N/A | N/A | N/A | N/A | N/A |
| **Renal and urinary disorders** | 0 (0.0) | 0 | 0.0 (0.0,0.0) | 1 (0.3) | 1 | 0.1 (0.0,0.7) | N/A | N/A | N/A | N/A | N/A | N/A |
| **Surgical and medical procedures** | 0 (0.0) | 0 | 0.0 (0.0,0.0) | 1 (0.3) | 1 | 0.1 (0.0,0.7) | N/A | N/A | N/A | N/A | N/A | N/A |
| n = Number of participants experiencing an event % = Percentage of participants experiencing an event Events = Number of adverse events IR = Incidence rate per 100 person-years 95% CI = 95% confidence interval of IR | | | | | | | | | | | | |
